# 

**Published:** 1893-01

**Authors:** 


					﻿CONTENTS:
ORIGINAL ARTICLES:	page
Some Texas Fever Experiments. By R. R. Dinwiddie.....................    1
Thermometry. By William Dougherty, Veterinarian........................ 14
Intussusception or Invagination. By J. F. Butterfield, V. S............ 17
SELECTIONS:
Is Scarlatina a Bovine Malady? By Professor McCall..................... 19
Diffuse Melanosis in a Cow. By Dr. Giuseppe Nuvoletti.................. 32
Veterinary Dental Surgery. By J. F. Fisher, V.S...’...................\.	36
EDITORIAL:
The Journal op Comparative Medicine and Veterinary Archives for 1898. 41
New York State Veterinary Medical Society.............................. 42
COMMUNICATIONS :
Answer to Dr. Shufeldt..............................................    44
Ontario Veterinary College Examinations..............................   46
University of Pennsylvania—Hospital for Dogs............................47
SOCIETY PROCEEDINGS:
Keystone Veterinary Medical Association...............................  49
Keystone Veterinary Medical Association................................ SO
Veterinary Medical Association of New Jersey........................... 58
Illinois State Veterinary Medical Association.......................... 56
Ontario Veterinary Medical Society..................................... 58
Faculty of Comparative Medicine and Veterinary Science..................59
Montreal Veterinary Medical Association.....'........................   61
Meeting of the Maryland State Veterinary Medical Society................68
Ontario Veterinary Association........................................  68
Massachusetts Veterinary Association....................................64
				

